# Ultrasound and Transcriptomics Identify a Differential Impact of Cisplatin and Histone Deacetylation on Tumor Structure and Microenvironment in a Patient-Derived In Vivo Model of Gastric Cancer

**DOI:** 10.3390/pharmaceutics13091485

**Published:** 2021-09-16

**Authors:** Aina Venkatasamy, Eric Guerin, Anais Blanchet, Christophe Orvain, Véronique Devignot, Matthieu Jung, Alain C. Jung, Marie-Pierre Chenard, Benoit Romain, Christian Gaiddon, Georg Mellitzer

**Affiliations:** 1 Streinth Lab (Stress Response and Innovative Therapies), Strasbourg University, Inserm UMR_S 1113 IRFAC (Interface Recherche Fondamental et Appliquée à la Cancérologie), 67200 Strasbourg, France; aina.vnkt@gmail.com (A.V.); eric.guerin@chru-strasbourg.fr (E.G.); anais.blanchet@gmail.com (A.B.); orvain@unistra.fr (C.O.); veronique.devignot@inserm.fr (V.D.); a.jung@icans.eu (A.C.J.); ben.romain@hotmail.fr (B.R.); 2 IHU-Strasbourg (Institut Hospitalo-Universitaire), 67091 Strasbourg, France; 3 IGBMC, GenomEast, 67400 Illkirch, France; jung@igbmc.fr; 4 Laboratoire de Biologie Tumorale, ICANS, 67200 Strasbourg, France; 5 Pathology Department, Hôpitaux Universitaires de Strasbourg, 67000 Strasbourg, France; Marie-Pierrette.CHENARD@chru-strasbourg.fr; 6 Digestive Surgery Department, Hôpitaux Universitaires de Strasbourg, 67000 Strasbourg, France

**Keywords:** gastric cancer, cisplatin, ultrasound, HDAC Inhibitors, SAHA, p53, microenvironment, transcriptomic, epigenetic, cancer immunity

## Abstract

The reasons behind the poor efficacy of transition metal-based chemotherapies (e.g., cisplatin) or targeted therapies (e.g., histone deacetylase inhibitors, HDACi) on gastric cancer (GC) remain elusive and recent studies suggested that the tumor microenvironment could contribute to the resistance. Hence, our objective was to gain information on the impact of cisplatin and the pan-HDACi SAHA (suberanilohydroxamic acid) on the tumor substructure and microenvironment of GC, by establishing patient-derived xenografts of GC and a combination of ultrasound, immunohistochemistry, and transcriptomics to analyze. The tumors responded partially to SAHA and cisplatin. An ultrasound gave more accurate tumor measures than a caliper. Importantly, an ultrasound allowed a noninvasive real-time access to the tumor substructure, showing differences between cisplatin and SAHA. These differences were confirmed by immunohistochemistry and transcriptomic analyses of the tumor microenvironment, identifying specific cell type signatures and transcription factor activation. For instance, cisplatin induced an “epithelial cell like” signature while SAHA favored a “mesenchymal cell like” one. Altogether, an ultrasound allowed a precise follow-up of the tumor progression while enabling a noninvasive real-time access to the tumor substructure. Combined with transcriptomics, our results underline the different intra-tumoral structural changes caused by both drugs that impact differently on the tumor microenvironment.

## 1. Introduction

Gastric cancer is the fifth most common cancer, and the third, in terms of mortality, worldwide, with a 5-year overall survival rate of 36% for operable cancers; this drops to 5–20% in locally-advanced/metastatic cancers, with a median survival of less than one year [1,2]. This highlights the need for progress in diagnosing and treating gastric cancer. Recent molecular classifications of gastric adenocarcinomas led to the identification of dysregulated pathways, at which targeted therapy attempts have been made, with unsatisfactory results [3]. Thus, the current standard therapies still rely on platinum-based drugs (cisplatin or oxaliplatin) that target DNA and induce the tumor suppressor gene p53, which, however is inactivated in more than 60% of gastric cancers [4,5,6]. Unfortunately, the survival rate of patients’ ongoing palliative therapies remains very low, as the majority (75%) of tumors are chemoresistant at late stages. The exact molecular basis of the resistance is not yet fully understood, despite the identification of several mechanisms, i.e., tissue-independent, such as the mutation in p53, the overexpression of DNA repair effector ERCC1 and the expression of ABC transporters, or tissue-specific [7,8]. In addition to altered/mutated intracellular mechanisms within the cancer cells, the tumoral microenvironment also plays an important role in the resistance to treatment by impacting the cancer cell survival via metabolites, mechanical stresses, and non-cancerous cells, such as cancer associated fibroblasts [9]. Furthermore, chemotherapies induce severe side effects on various tissues, including kidneys, the nervous system, and the muscles [10,11]. These side effects also impact the therapy response. For instance, muscle atrophy has been associated with a poor response to chemotherapy in several studies, although this fact is still debated [12]. Hence, cancer should not be considered a localized pathology, but rather a systemic disease involving a complex ecosystem composed of the cancer cells, the microenvironment, and distant tissues, all impacted and directly/indirectly involved in the response to treatment.

Amongst the mechanisms impacting the response to therapy, increased expression of several histone deacetylases (HDACs) has been described in gastric cancers, such as HDAC4 [13]. HDACs are important regulators of the epigenetic and transcriptional mechanisms that control gene expression in eukaryotic cells [14]. In addition, HDACs can also remove acetyl residues from various transcription factors that are key in gastric carcinogenesis and tumor aggressivity, such as p53 and HIF1A [15]. However, the targeting of HDACs using the pan-HDAC inhibitor Vorinostat (SAHA) showed no gain in gastric cancer patient survival, when administrated without patient stratification [16]. The reason for this lack of efficacy in patients remains undocumented, highlighting the necessity for additional investigations that focus on possible intra-tumoral clonal variation and/or the influence of the tumor microenvironment. A precise analysis of the molecular and cellular basis of SAHA antitumor activity might lead to better rationalization of the targeting of HDACs in cancer and the use of novel treatment combinations that might improve the therapeutic care.

Currently, in translational cancer research, patient-derived xenograft (PDX) models are privileged tools for anti-cancer drug testing, as they retain key histological, molecular, and genetic characteristics of the primary tumor [1,17,18,19,20]. Therefore, PDX models allow time-course follow-up of tumor progression and repeated measures, in response to various conditions, together with the analysis of intra-tumor clonal variations [19]. To our knowledge, the anticancer activity of SAHA has never been tested and compared to platinum-based chemotherapy on a gastric cancer PDX model yet, likely because of the low success rate for the establishment of such models (around 25%) [18,20,21,22]. Hence, to further characterize the anticancer properties of SAHA and the consequences of HDAC inhibition on gastric cancer aggressivity, we decided to develop and use a PDX model of gastric cancer. In particular, we wanted to assess some of the changes in the intra-tumoral structure and microenvironment of the tumors induced by cisplatin or SAHA.

Measurements of the tumor burden are generally performed using manual calipers to evaluate the in vivo cytostatic and cytotoxic activity of anticancer drugs in mouse models [5,18,21,22,23]. If this measurement technique is quick and easy to perform, it also bears several important limitations, namely the inter-user-based variability, the difficulty to measure a third dimension, and the restriction to an external/superficial visualization of the tumor aspects. In this respect, non-invasive imaging techniques, such as magnetic resonance imaging (MRI) or micro-CT (small animal computed tomography), are superior to calipers, but require a long and complex setup with higher costs and, therefore, are more difficult to apply to large groups of animals [24,25]. For these reasons, to measure the effect of SAHA on tumor growth comparatively to standard chemotherapy, we decided to use an ultrasound, enabling direct access to external (i.e., volume) and internal (i.e., structure and morphology) treatment-related changes, in addition to several other advantages. For instance, the tumor growth could be followed over time with recorded images, measurable by multiple experimenters. Additionally, an ultrasound allows a direct access to the tumor internal structure and vascularization [19,25,26,27,28]. This appears particularly relevant with regard to PDX models, since their primary interest is to closely reproduce the structural complexity of the patient’s tumor tissue. 

Hence, our objectives were: (1) to evaluate the efficacy of the inhibition of HDACs by suberoylanilide hydroxamic acid (SAHA), comparatively to cisplatin on tumor growth in a PDX model of gastric cancer, by comparing manual caliper measurements to ultrasound measurements; (2) analyze the ultrasound images for changes in tumor morphology over time; (3) and use immunohistochemistry analyzes and RNA sequencing to further characterize the impact the different treatments have on the tumor ecosystem. Our results showed that ultrasounds pinpointed to differences in internal tumor structures caused by SAHA and cisplatin, which we validated using immunohistology analyses and transcriptomics/bioinformatics clustering analyses of the murine tumor microenvironment.

## 2. Materials and Methods

### 2.1. Manual (Mechanical) Vernier Caliper Measurement and Ultrasound Evaluation

**Figure 1:**
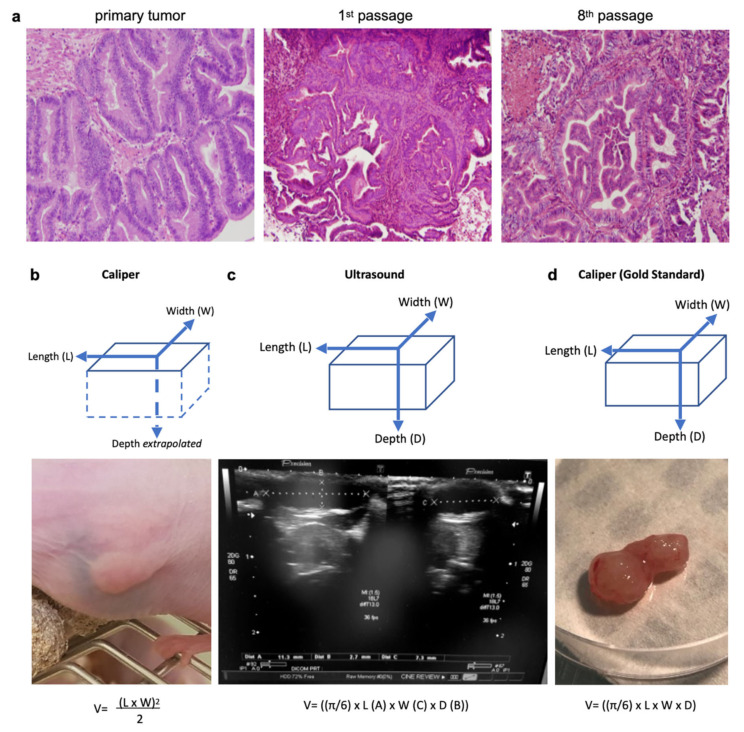
Three measurement techniques on primary gastric adenocarcinoma tumors grown in nude mice (PDX): caliper and ultrasound on living mice, and caliper on ex vivo tumors (gold standard). (**a**) Histological hematoxylin eosin staining (magnification 40×) of the primary human tumor compared to tumors issued from it grown in a PDX mouse (1st and 8th passage). The PDX tumors retained the major architectural and histological traits of the primary tumor and maintained across passages from mouse-to-mouse. (**b**) The caliper on the live mouse only measured the length and width, and the depth was estimated from the width. (**c**) An ultrasound enabled the measurement of all three dimensions of the tumor (length, width, and depth). (**d**) The caliper on the ex vivo tumor was the gold standard for length, width, and depth measures. Formulas used for tumor volumes are indicated below.

A caliper gave a two-dimensional measure (length and width) and the thickness of the tumor was extrapolated from the width. The volume was calculated as such: V = (length × width)^2^/2 (Figure 1b) [23]. An ultrasound was performed using B-mode images on a Toshiba Ultrasound Aplio XG (SSA-790A) (Toshiba, Tochigi, Japan) with a superficial transducer (probe frequency 14 MHz), which has an axial spatial resolution of 0.11 mm. An ultrasound, based on acoustic echoes from high-frequency acoustic waves, measured all three dimensions of the tumor (length, width and thickness) (Figure 1c), and the volume was calculated using V = [(π/6) × length × width × thickness] [29]. With an ultrasound, we also visually assessed the tumor morphologies: (i) the presence of solid echoic components; (ii) the presence of cysts (>2 mm); (iii) the location of the cysts (central or peripheral); and (iv) the presence of hyperechoic images.

After the final caliper/ultrasound measurements, the tumor was excised for ex vivo macroscopic evaluation. All three dimensions of the tumor (length, width, and thickness) were measured using the same manual caliper and served as the gold standard (Figure 1d). The volume was calculated with the same formula as for the ultrasound: V = [(π/6) × length × width × thickness]. Overestimation of the tumor size occurred when the measured volume was >10% compared to the same gold standard volume.

### 2.2. Histological Evaluation

After the final caliper/ultrasound measurements, the tumor was excised for ex vivo histological and immunohistological evaluation. All tumors were cut in halves along their greater axis and fixed in 4% paraformaldehyde solution for hematoxylin-eosin (H&E) staining. All slices were read at ×2–×20–×40 magnifications, blinded to the treatment group. The following elements were analyzed: (i) necrosis and intraglandular necrosis; (ii) cysts; (iii) location of the cysts (central or peripheral); (iv) stroma; and (v) glandular papillary tumoral structures.

### 2.3. Immunohistochemistry (IHC)

IHC was performed on deparaffinized histological tumor slices (5 µm). Unmasking was carried out with Na-citrate in the microwave oven (800 W, 10 min). After washing once with distilled water and once with PBS, the sections were permeabilized with 0.1% PBS-Triton for 5 min and the nonspecific sites were blocked in 5% NGS-PBS-Triton 0.1% (normal goat serum) for 60 min. Subsequently, primary antibodies to P53 DO1 (SC126, 1/200, Santa Cruz^®,^ Dallas, TX, USA), H3K9 (NB22-0122, 1/300, Neobiotech^®^, Seoul, Korea), Ki67 (sp6) (RM-9106.S, 1/500, Thermo Fisher RM, Waltham, MA, USA) and α-actin (aSMA, 1/500) were diluted in PBS + Triton 0.1%, then added and kept at 4 °C overnight. After 3 washes with PBS-Triton 0.1%, slides were incubated with the secondary antibody (diluted 1:1000 in 1×PBS/5% NGS) specific to the primary antibody, for 1 h at room temperature. Slides were mounted with FluorSave Reagent Mounting Medium (Calbiochem^®^, San Diego, CA, USA) and allowed to dry overnight before observation using a fluorescence microscope (Axio Imager 2—ZEISS, Oberkochen, Germany). Positive staining was assessed based on negative control (e.g., no primary antibody). Images were taken with consistent time of exposure and gain between conditions. Images were then analyzed by Fiji software for quantification, using identical settings between conditions. Quantifications were performed by measuring the number of pixels for each intensity of the white signal, ranging from 0 (dark) to 255 (white).

### 2.4. Transcriptomic Analysis

RNA was extracted from PDX-tumors using TRIzol-mediated cell lysis. After extraction and RNA precipitation, supernatants were removed and the RNA pellet was washed with 75% EtOH, centrifuged at 9000× *g* for 5 min at 4 °C, and again, 75% EtOH was added. Then, the RNA was resuspended in nuclease-free H2O and quantified using NanoDrop Spectrophotometer (Thermo Scientific, Waltham, MA, USA).

To identify the deregulated genes in the mouse microenvironment of the tumor, RNA-Seq was performed on extracted total RNAs and the sequences obtained were selectively aligned on a chimeric genome composed of the mouse genome (mm10) and human genome (hg38) using STAR version 2.5.3a. Quantification step was performed using HTSeq-count version 0.6.1p1, with annotations from Ensembl version 103 (Homo sapiens) and 102 (Mus musculus), and then data were further processed with AltAnalyze version 2. After standard normalization, deregulated genes with log2 fold change >1.5 and adjusted *p*-value <0.05 were selected and pathways enrichment analyses were performed using multiple databases (e.g., DAVID, STRING, Reactome, TRAP, Biomarkers).

## 3. Results

### 3.1. The Pan-HDAC Inhibitor SAHA and Cisplatin Reduce Tumor Growth in a Gastric Cancer PDX Model

To establish the gastric cancer PDX model GCX-004, a surgical specimen was received, with limited ex vivo time (<25 min), after total gastrectomy of a primary well-differentiated intestinal type HER2-negative gastric adenocarcinoma (ypT1aN0) from a 64 year-old man treated with preoperative chemotherapy (EOX: epirubicin + cisplatin + 5-fluoro-uracil). The tumor fragments (3 *×* 3 mm) were implanted on the flanks of an anaesthetized immunodeficient mouse (Supplementary Material Figure S1a), following close monitoring of their growth (1–3 times/week by caliper). When the implanted tumor reached 500 mm^3^, the engraftment was considered successful, when the tumor reached ~1000 mm^3^ the mouse was anaesthetized, and the tumor harvested for serial transplantation into successive mice generations. Histology of the different passages of GCX-004 were well-matched with the primary human tumor (Figure 1a) without lymphomatous transformation. In addition to the histological stability over time and passages, the PDX retained the genetic characteristics of the primary tumor, with no potentially pathogenic actionable mutations on the NGS panel of 26 clinically-targetable genes (Figure S1b). The tumor harbored a p53 mutation: exon8:c.844C > T:p.R282W.

**Figure 2:**
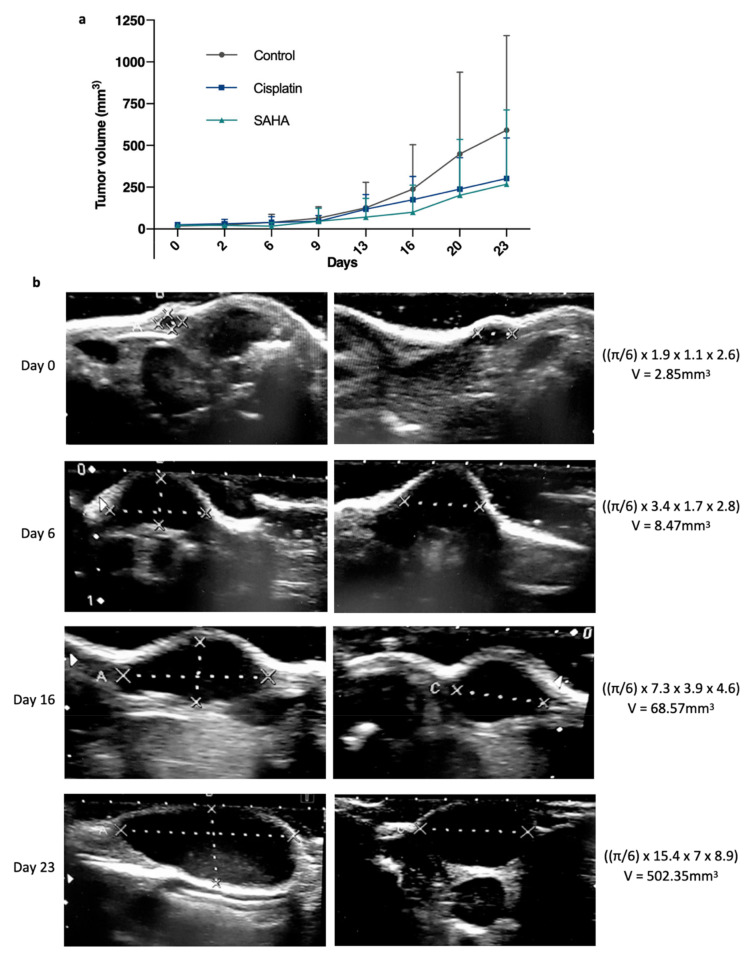
Evolution of the gastric tumor growth between treated and non-treated tumors. (**a**) Average tumor volume growth over time, measured by a manual caliper on living mice from day 0 to day 23, between suberanilohydroxamic acid (SAHA, HDAC inhibitor), standard chemotherapy using cisplatin, which targets DNA, and a control group (*n* = 12, 12, 8). *p* < 0.05 between control and treated groups as established by a *t*-test. (**b**) Illustration of representative ultrasound images and calculations showing the evolution of a GCX-004 tumor from the control group over time (day 0, day 6, day 16, and day 23).

To compare the anticancer efficacy of cisplatin with the pan-HDAC inhibitor SAHA, several tumor fragments (~3 × 3 mm) from GCX-004 (passage 8) were implanted subcutaneously on the flanks of 16 athymic NUDE mice. Then, the mice were split into three groups. Overall, no significant growth difference between the left and right implanted sides were observed, except in two cases where the tumor did not develop further and we decided to remove these mice from the cohort. Once the tumors reached the target volume (100—150 mm^3^), we treated one group (*n* = 6) with cisplatin (5 mg/kg body weight), a second one (*n* = 4) with Vorinostat (SAHA 50 mg/kg body weight) and the control group (*n* = 6) with DMSO-PBS-cremophor. The volumes were repeatedly measured over time using a caliper and an ultrasound (every 3–4 days, Figure 1b,c). At day 23, final measurements were performed, tumors resected, and re-measured by the caliper (gold standard) (Figure 1d). The dissected tumor volumes ranged from 29 to 1444 mm^3^. The smallest tumor measured V = [(π/6) 7 × (length) 4 × (width) 2 × (thickness)] = 29 mm^3^, corresponding to approximately 7,000,000 cells (average cell diameter of 20 µm). Ultrasound measures showed that cisplatin-treated tumors (*n* = 12; 239.8mm^3^ ± 159) were smaller than the controls (*n* = 12; 561.5mm^3^ ± 138.5) (*p* = 0.0382) (Figure 2a). SAHA-treated tumors (*n* = 8; 229.6mm^3^ ± 466.2) were also smaller than the controls (*p* = 0.6349). Typical ultrasound images of the evolution of one tumor from the control group are represented in Figure 2b.

### 3.2. Ultrasound Outclasses Caliper for Precision Measurement

**Figure 3:**
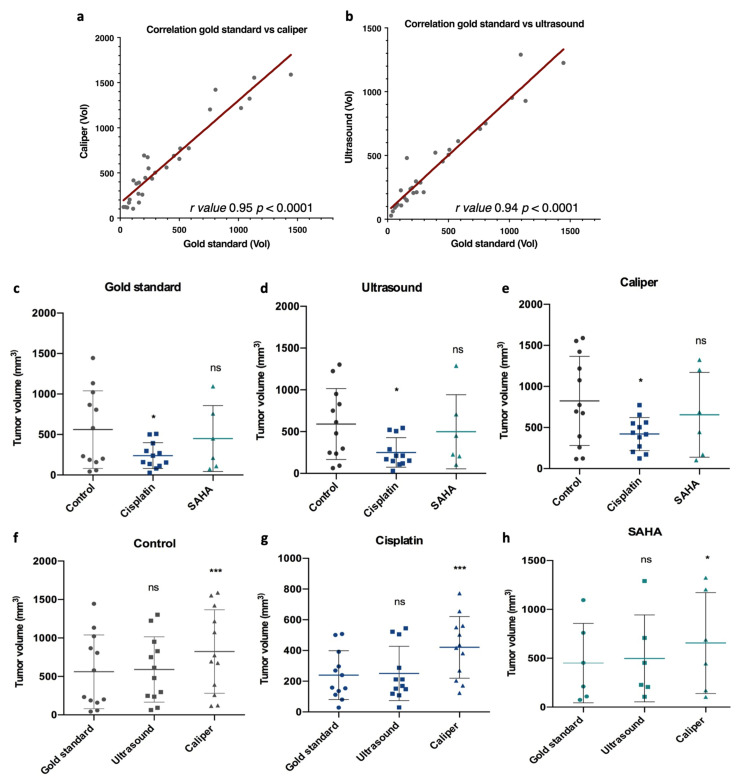
Correlation and comparison of the tumor volume among the gold standard, caliper, and ultrasound measurements, and for each treatment group (control, cisplatin and SAHA). At day 23, final tumor volumes were measured and compared for each measurement method. (**a**,**b**) Gold standard measurements were correlated with caliper or ultrasound measurements using GraphPad Prism, with r values 0.95 and 0.94, respectively, and with a *p* value < 0.0001. Then measurements were compared among each treatment group for the gold standard (**c**), caliper (**d**), and ultrasound (**e**), and for each treatment group among the different measurement methods, control (**f**), cisplatin (**g**), and SAHA (**h**). Control *n* = 12, cisplatin *n* = 12, SAHA *n* = 6. Data were subjected to statistical analyses using GraphPad software version 6.0 (ns = not significant, * = *p* < 0.05, *** = *p* < 0.001).

To compare the performances of an ultrasound to standard caliper measures, we removed from our study tumors that disappeared upon treatment (two tumors in the SAHA group), as, obviously, no comparison was possible. Using this set of tumors, we first performed correlation analyses, comparing the gold standard to the caliper or ultrasound, which showed a good r in both cases, 0.95 and 0.94, respectively (Figure 3a,b). The r was slightly better for the caliper, likely due to one ultrasound measure that was significantly overestimated compared to the gold standard; excluding this specific case, we obtained r = 0.9584 for the ultrasound and r = 0.9520 for the caliper. Reanalyzing this specific case showed that this tumor was the longest and largest one in the cohort and, therefore, perhaps more difficult to measure.

Then, we analyzed the performances of each measurement method according to the treatment group. We compared the measures taken by the caliper after dissection (gold standard, Figure 1d), by the caliper before dissection (Figure 1b), and by the ultrasound before dissection (Figure 1c). The first interesting observation was that ultrasound measures gave a better statistical difference than caliper measures (*p* = 0.018 and *p* = 0.0243 respectively) when comparing the control group and the cisplatin group, highlighting the relevance of an ultrasound to evaluate tumor growth (Figure 3d,e). Importantly, with this set of tumors, no statistical differences were observed between the control and SAHA group. In addition, we observed that ultrasound and gold standard measurements showed a similar final tumor volume profile among the control, cisplatin, and SAHA-treated groups, as indicated by *p* values above 0.05 (Figure 3c,d,f,h). For instance, in the control group, there was no significant difference between the gold standard (561.5 ± 479.7 mm^3^) and ultrasound measures (590.9 ± 425.6 mm^3^, *p* > 0.05) (Figure 3f). In contrast, the caliper showed a significantly higher final volume compared to the gold standard and, therefore, the ultrasound (*p* < 0.0001), regardless of the treatment group (Figure 3e,h). For instance, in the control group, there was a statistically significant difference between the caliper and gold standard measures (*p* < 0.001, Figure 3e). The same was also observed with cisplatin (*p* < 0.0001, Figure 3g) and SAHA (*p* < 0.05, Figure 3h). Overall, the tumors overestimated by the caliper were, on average, smaller (368.9 mm^3^) than those it underestimated (573.8 mm^3^). Overall, the ultrasound performed better than the caliper and volume overestimation (>10%) was less frequent (38%).

### 3.3. Ultrasound Identifies Architectural Differences in Cisplatin and the Pan-HDACi Treated Tumors

**Figure 4:**
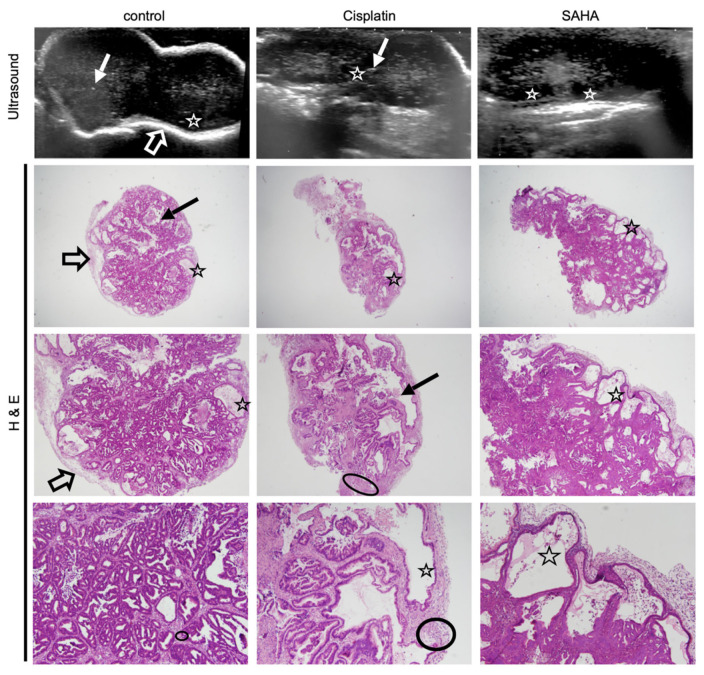
Histology confirms treatment-related sub-structural changes observed with ultrasound. Ultrasound images of a representative GXC-004 tumor from each treatment group (upper panel, day 23) compared to their corresponding histological slices (hematoxylin eosin staining, magnification ×2 (upper), ×20 (middle) and ×40 (lower)). Control tumors had sharper margins with a white peripheral rim (empty arrow), while treated tumors, especially SAHA-treated ones, were more lobulated. Some tumors (especially in the control group) presented scarce intraglandular necrosis, which could potentially explain the comet-tail artifacts observed on ultrasound (i.e., small central hyperechoic punctiform foci, plain arrow). Histology revealed a stromal predominance (circled) in cisplatin-treated tumors compared to the primary tumor and controls, with a stroma/gland ratio > 50%. Cysts (star) were frequently observed with an ultrasound (visible when >2 mm) in the periphery of SAHA-treated tumors, less frequently in cisplatin-treated tumors. This finding was confirmed by histology (star).

In addition to post-therapeutic modifications of tumor volume, the ultrasound also showed structural changes between treated and control tumors (Figure 4). For instance, some tumors appeared more solid, with small central hyperechoic punctiform foci with “comet-tail” artifacts (plain arrow in Figure 4, left and center panel), best seen in control tumors and potentially related to focal areas of scarce intraglandular necrosis. Other tumors, especially SAHA-treated ones, appeared more cystic, presenting small round peripheral anechoic cysts (clearly visible when >2 mm) in the periphery (stars in Figure 4).

**Figure 5:**
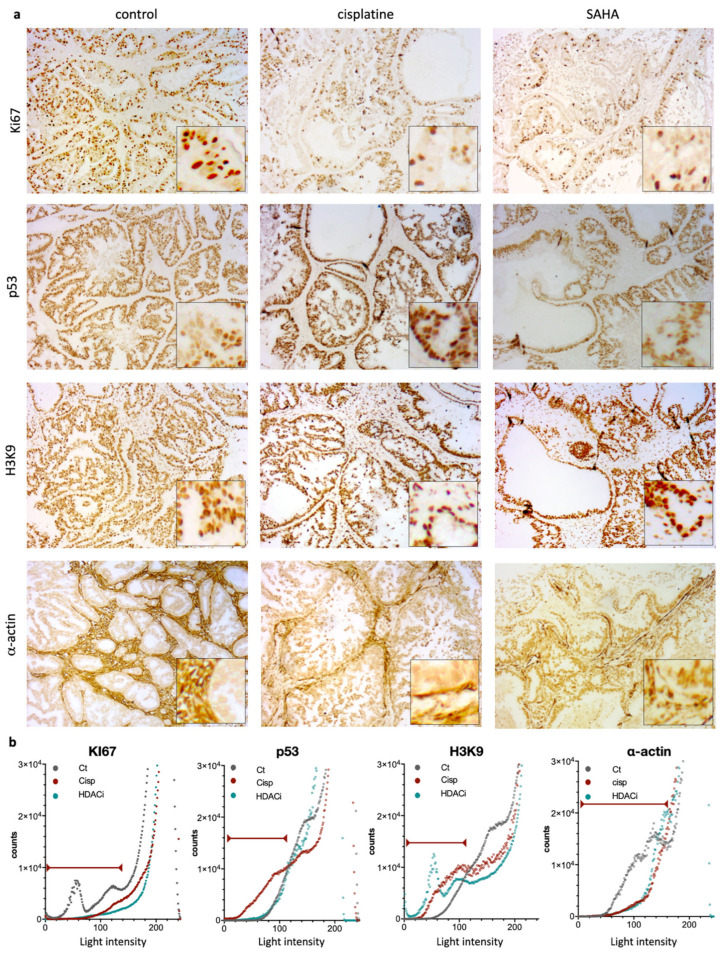
Immunohistochemical (IHC) staining and intensity measures for Ki67, p53, H3K9, and α-actin. (**a**) Representative photomicrographs of the expression of Ki67, p53, H3K9, and α-actin for treatment group (control, cisplatin, and SAHA (magnification ×20 with close up ×40). (**b**) Quantifications by Fiji software of each condition. Graphs represent number of pixels for each light intensity from 0 to 255. Red lines indicate the relevant values as considered, based on negative control (e.g., no primary antibody).

This suggested potential differences regarding the underlying tumor composition and architecture. To confirm this, we performed a histological analysis using hematoxylin eosin staining (Figure 4, H&E panels). We also investigated the expression of alpha-actin, a marker selective for cancer-associated fibroblasts (CAF, α-actin, Figure 5). CAFs are present in the tumor microenvironment at various levels and contribute to the tumor aggressivity and structure [28]. To verify the impact of cisplatin and the pan-HDACi SAHA on gastric cancer cells, we also analyzed the expression of Ki67, p53, and the acetylation of the histone H3 at the lysine 9 position (H3K9), which are markers for cell proliferation, DNA damage, and a target of HDACs, respectively (Figure 5).

When considering the control group, all tumors appeared homogenous on ultrasound, with well-defined borders (confirmed during macroscopic examination) and a peripheral hyperechoic (white) rim, also observed on histology (Figure 4, control, “thick arrow”). The tumors were predominantly solid (hypoechoic), with no significant histological architectural changes compared to the human primary tumor. The few scattered small anechoic cysts observed in the periphery of the tumor were also confirmed by histology (indicated by stars in Figure 4). Similarly, some tumors presented with scarce intraglandular necrosis, which could be an explanation for the comet-tail artifacts observed on the ultrasound (small central hyperechoic punctiform foci indicated by plain arrows, control; Figure 4). More than 50% of the tumor cells presented positive nuclear staining for Ki67 (Figure 5a). IHC showed positive nuclear staining for p53 in cancer cells, but no apparent p53 expression was detected in the non-neoplastic tissue. Expression of p53 in cancer cells in the control condition is coherent with the presence of the R282W mutation in p53 in the GCX-004 PDX model, detected by NGS, as p53 mutants are known to display elevated protein levels in cancer cells. In contrast, untreated tumors showed positive nuclei staining for acetylated histone at lysine 9 (H3K9), both in the cancer cells and the cells of the microenvironment. In addition, the tumor tissue showed a strong and dense expression of α-actin in the microenvironment, suggesting a dominant presence of CAFs. Interestingly, there was no penetration of CAFs within the organized intestinal-type glands of the gastric cancer cells.

On the ultrasound, all cisplatin-treated tumors had slightly less defined lobulated borders (slightly irregular external surface on macroscopic examination) with no peripheral (white) rim, unlike control tumors (Figure 4, empty arrows). The tumors showed solid hypoechoic central portions with round anechoic peripheral cysts (Figure 5, stars), which were more frequent than in the controls and confirmed on histological slices. Histology also revealed a stromal predominance (Figure 4, circles) compared to the primary tumor and controls, with a stroma/gland ratio > 50%. Thus, the tumor burden (cancer cells/mm^2^) was lower. Regarding morphological changes, the stroma appeared hypoechoic (dark grey) on ultrasound with a similar echogenicity (i.e., appearance on ultrasound) to the tumor tissue, explaining why the ultrasound could not differentiate stroma from glandular tissue. Interestingly, the stroma dominance was associated with a decrease in the alpha-actin staining and a disorganization of the CAFs in the microenvironment, suggesting a direct or indirect impact of cisplatin on CAFs (Figure 5a,b). As expected, cisplatin treatment resulted in a strong decrease of Ki67, but an increase in p53 expression (Figure 5a,b). Interestingly, cisplatin significantly increased the staining of H3K9 in the cancer cells, as well as in the stroma, highlighting the impact of this chemotherapy on epigenetic mechanisms in tumors (Figure 5a,b). Ultrasound images also showed that all SAHA-treated tumors had less defined slightly lobulated borders and were far more cystic (and therefore more hypoechoic) than cisplatin-treated ones or controls, with a peripheral rim of small anechoic cysts and a solid hypoechoic central portion (Figure 4, stars). This lobulated aspect, together with the presence of a rim of peripheral cysts, was confirmed by the macroscopic evaluation, with partially cystic and very lobulated tumors. Histology confirmed these findings, showing a rim of peripheral monostratified cysts (Figure 4, stars) and a glandular central portion of the tumor (i.e., no stromal predominance, Figure 5). As observed for cisplatin, the inhibition of HDACs by SAHA significantly diminished alpha-actin staining, without effecting CAFs’ organization (Figure 5a,b). As expected, Ki67 staining was significantly reduced and the acetylation of lysine 9 of histone 3 (H3K9) was strongly increased in cancer cells and the stroma.

The correlation between the ultrasound images and the immunohistological observations indicates that ultrasound can pinpoint changes in the tumor internal structure, as well as changes in the stroma/microenvironment. These analyses also indicate that cisplatin and SAHA reduce cancer cell proliferation in gastric tumors, but their respective impact on the tumor stroma/microenvironment seemed different.

### 3.4. Transcriptomics Analyses Confirm Differences in the Mouse Stroma/Microenvironment of the Human Gastric Tumor Induced by Cisplatin and the Pan-HDACi

**Figure 6:**
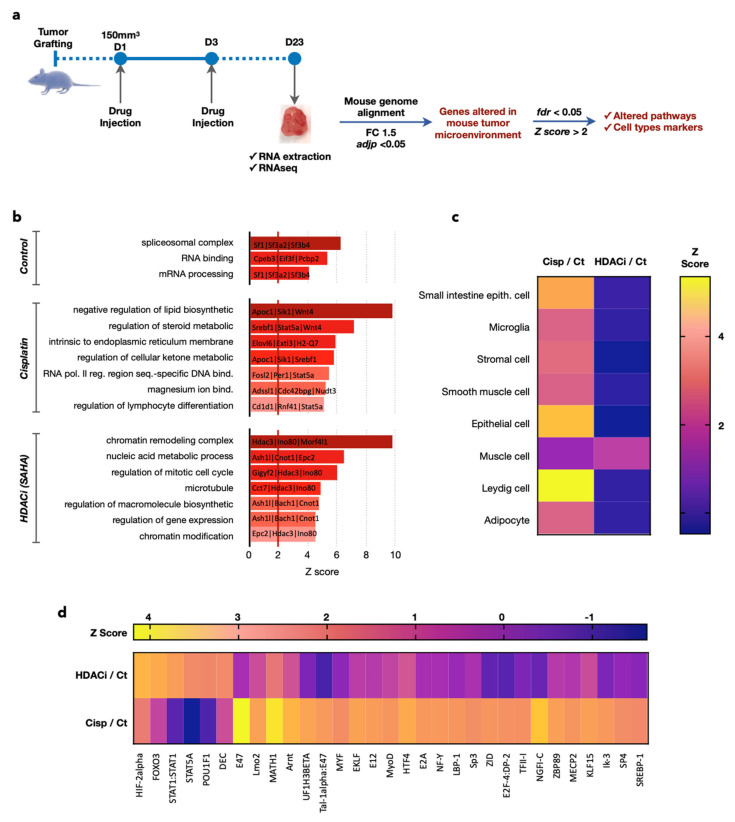
Transcriptomics analyses confirm differences in the mouse stroma/microenvironment of the human gastric tumor caused by cisplatin and SAHA. (**a**) Total RNAs were extracted from the tumors. Sequences obtained were specifically aligned on the mouse genome. Deregulated genes with fold change >1.5 and *p*-value < 0.05 were analyzed. False discovery error <0.05 and Z score above 2 were chosen as the cut-off in bioinformatics analyses and AltAnalyze was used to find gene signatures corresponding to given processes representing biomarkers selective of each condition (**b**), typical signatures for specific cell types (**c**), regulation of specific transcription factors (**d**).

Instead of testing numerous additional stromal markers to confirm the differences in the stroma structures observed with ultrasound and IHC, we took advantage of the RNA-Seq analyses (Figure 6a). The sequences obtained were specifically aligned on the mouse genome, allowing us to analyze the mouse microenvironment in PDX gastric tumors in response to the different treatments. After normalization, deregulated genes were selected according to an absolute value of a log2 fold change > 1.5 and an adjusted *p*-value < 0.05. The list of resulting genes was then used to identify altered pathways and specific makers in each experimental condition. False discovery error < 0.05 and Z-score > 2 were chosen as cut-off. For instance, clustering analysis identified genes corresponding to given processes representing biomarkers selective of each condition, such as spliceosome complex, negative regulation of lipid biosynthetic, and chromatin remodeling complex for control, cisplatin, and SAHA treatment, respectively (Figure 6b, Supplementary Material Figure S1c).

Clustering analyses also identified typical signatures for specific cell types that were induced either with cisplatin or SAHA (Figure 6c). Strikingly, cisplatin induces the most diverse signatures for various cell types of epithelial and mesenchymal phenotype (e.g., adipocytes, Leydig cells, stromal cells). In contrast, SAHA only induced one muscle related signature, but the magnitude of this dysregulation was higher than the one observed upon cisplatin treatment.

Obviously, the induction of these signatures does not mean that these cell types are present in the stroma of the PDX tumors, they are just an indication of the differential impact of cisplatin and SAHA on the gene expression of the mouse cells present in the stroma/microenvironment of the human gastric tumor grown in the PDX model. However, the induction of signatures corresponding to epithelial, stromal, microglia, and/or adipocyte cells might correspond to the recruitment of these cells into the vicinity of the tumor. The analysis of the changes in the gene expression also pointed out that the activity of different transcription factors was altered depending on the treatment (Figure 6d). For instance, E47 and MATH1 were the most induced by the treatment with cisplatin, while HIF-2 and FOXO3 were preferentially induced by SAHA.

Taken together, the transcriptomic analyses confirmed the initial ultrasound-based observation that cisplatin and the pan-HDACi SAHA impact on the mouse microenvironment and, hence, modified the internal tumor structure and microenvironment.

## 4. Discussion

Cisplatin is widely used to treat cancers, including gastric ones, despite the existence of inherent poor response in some cases, mostly related to the development of resistance mechanisms, which are not completely understood. As an alternative, pan-HDACi have been developed and showed promising response in standard xenografts models of gastric cancer cell lines, which have, unfortunately, not been confirmed during phase 2 clinical trials. Hence, to gain further understanding of the impact of cisplatin and pan-HDACi in gastric cancer, we compared their activity on a patient-derived xenograft model of gastric cancer, by focusing our interest on a real-time longitudinal analysis of the intra-tumoral structural changes using ultrasound, immunohistochemistry, and a transcriptomic analysis of the murine microenvironment of the tumors.

### 4.1. Cisplatin Chemotherapy and the Pan-HDACi SAHA Reduce Tumor Size in a Patient-Derived Xenografts Model of Gastric Cancer

In preclinical translational studies, one of the most relevant approaches to assess the outcome of a treatment is through the tumor volume and its speed of growth using different mouse models [30]. The most commonly used mouse models are either cell line-derived models or patient-derived xenografts (PDX). Cell line-derived models are overwhelmingly represented in the literature (82% of studies until 2016), but they raised an increasing number of criticisms for their distorted tissue architecture, with altered microenvironment and lack of genetic heterogeneity [22]. On the contrary, PDX models (7% of recent studies) remain stable across many generations and retain the major traits of their originating tumor; thus, resulting in the only model able to reflect the vast patient and tumor variability and heterogeneity [27]. We decided to use such a PDX model to comparatively analyze the impact of cisplatin to the effect of SAHA, an inhibitor of HDACs, on gastric tumor growth. Our goal was to find clues that may explain why SAHA did not succeed in gastric cancer patients’ clinical trials and that could provide direction for future improvements. The PDX model used in this study displays typical features of gastric cancer of the intestinal subgroup. As such, the tumors present a glandular, intestinal-type organization, which is not observed with cell line xenografts. The conservation of such architectural organization means that the impact of the drugs on cancer cells, as well as their microenvironment, is more likely to mirror what is happening in the ‘real’ human tumor. In this respect, the use of PDX allowed us to observe that cisplatin and SAHA reduced tumor size in this PDX model of GC. Interestingly, it also allowed visualizing the structural changes in the “glandular” organization of the tumors following cisplatin and SAHA treatment, such as apparition of vacuoles on various extents. However, the treatment did not profoundly and homogeneously affect the glandular structure, suggesting that the molecular impact of the treatments did not modify the differentiated phenotype of the cancer cells.

### 4.2. Ultrasound Is More Precise Than Caliper to Measure Changes in Tumor Growth

The measurements of tumor size obtained with the ultrasound were more precise than the ones obtained with the caliper. A similar conclusion was previously drawn by Ayers et al. when they showed that ultrasound-based measures of the tumor volume were more accurate and reproducible than caliper ones on non-treated cell-line derived xenografts tumors [23]. However, nothing has been published so far about ultrasound measurements of patient-derived xenografts or chemotherapy-treated tumors. Our results show that caliper measurements appear to overestimate the tumor volume, partially due to the calculation method and the lack of measures for the tumor depth. Other explanations for this overestimation are: dermal or subdermal skin layer interpositions, irregular non-ellipsoid tumors, and dependency on the user skills. Similar conclusions were driven from cell-line derived xenografts [23,25]. However, the caliper remains extensively used to perform classical tumor-volume measurements, as it is inexpensive and easy to manipulate, despite poor inter-observer reproducibility and accuracy [25,26]. Importantly, in our gastric tumor PDX model, there was no statistically significant difference between ultrasound measures on live mouse and “gold standard” ones on the ex vivo tumor, which is in agreement with what Ayers et al. observed. This accuracy is likely due to the fact that the tumor implantation site on the flanks of the mouse (i.e., heterotopic implantation) is well suited for ultrasound imaging with a high-frequency probe, as the explored tumors are superficial (i.e., subcutaneous) and not subject to bone/air artifacts. With the ultrasound, the major limiting factor is the depth of the tissue that can be imaged through, especially when using superficial probes [30]. If the ultrasound is limited by its user’s skills, the exploration of superficial tumors is also not limited by the interposition of skin layers or by non-ellipsoid tumor shapes, therefore enabling a real-time visualization of the tumor, together with the storage of images of interest. However, fitting very long and/or large tumors (volume 1500–2000 mm^3^) reaching the endpoint of the experimental procedure, into the probe’s window of exploration, may be a bit more technically challenging.

### 4.3. Cisplatin and the Pan-HDACi SAHA Induce Changes in Intratumoral Substructures That Can Be Detected by Ultrasound

In addition to being an efficient measuring device, the ultrasound also provides direct access to the tumor morphology. In humans, the ultrasound has been used to characterize and differentiate between small superficial (i.e., cutaneous or subcutaneous) soft-tissue tumors (e.g., melanoma, angiosarcoma, lipoma, epidermoid cysts, nerve sheath tumors, etc.), which, from their locations, are comparable to our subcutaneous PDX tumors [31,32]. On human subcutaneous tumors, it enabled real-time access to the tumor structure, prior to the biopsy or surgical removal [32]. In addition to the characterization of tumors, the real-time access to the tumor architecture may also lead to the observation of treatment-related structural changes. With the development of novel targeted therapies, especially immunotherapies, the size and volume of tumors alone are now insufficient to judge the efficacy of such treatments [33]. For instance, swelling and/or necrotic changes have been described in tumors treated by immunotherapy, forcing us to move away from the caliper and size measures alone and, therefore, favoring real-time imaging evaluation of novel drugs efficacy [33,34]. For that purpose, the ultrasound appeared as an interesting alternative, enabling real-time visualization of structural changes within tumors. In our study, we observed the appearance of multiple small cysts in treated tumors (especially with SAHA), which were already visible on live mice ultrasound when >2 mm, later confirmed by histology. On human ex vivo lymph nodes, Saegusa-Beecroft et al. estimated that the spatial resolution of the ultrasound probe was sufficient to image 0.2 to 2 mm-wide micro metastasis, compared to histology as the gold standard, which is in agreement with the spatial resolution of our probe (0.11 mm) [35]. If the ultrasound evaluation is currently not the standard-of-care follow-up procedure of human superficial tumors, it has been used to observe intra-tumor chemotherapy-related changes in women with breast tumors [36,37]. Regarding the architectural analysis of tumors, alternative imaging approaches exist (e.g., micro-CT, PET-CT or MRI) [14,20,38,39,40,41,42]. However, such methods, despite being more precise, are far more time-consuming, thus limiting the number of animals that can routinely be analyzed. Additionally, they require well-trained operators to run the equipment and represent a higher financial burden. Therefore, even if CT and MRI present higher spatial resolution and PET-CT permits a more functional approach, the caliper and ultrasound remain better suited for an easy-to-follow, real-time assessment of the tumor volume and growth, while limiting the costs of such a day-to-day evaluation [23,25,42].

### 4.4. Cisplatin and Pan-HDAC Inhibitor Cause Different Impact on the Tumor Microenvironment

The ultrasound has proven useful in image volume changes and/or structural modifications on live mice. If histology confirms what we observed on the ultrasound, underlying biomolecular changes are also observed. The decrease in tumor volume and of Ki67 positivity of tumor cells are reliable markers of drug efficacy, especially regarding their cytotoxic activity [43]. One of the cytotoxic mechanisms of platinum-based compounds relies on the activation of the p53-dependent apoptosis, as the *p53* gene plays a critical role in cellular response to DNA damage [44,45]. Consequently, the absence of a wild type p53 function results in resistance to anticancer agents, such as cisplatin. In gastric cancers, overexpression of p53 is strongly linked to its mutational status, also correlating to resistance to cisplatin [6,7,8,9,10,11,12,13,14,15,16,17,18,19,20,21,22,23,24,25,26,27,28,29,30,31,32,33,34,35,36,37,38,39,40,41,42,43,44,45,46]. In our PDX model, diffuse and homogenous p53 nuclear staining was observed in controls, related to the p53 mutation (exon8:c.844C > T:p.R282W). Despite the p53 mutation, cisplatin can induce the p53 protein level, in contrast to SAHA, which does not change it. The presence of a p53-mutation in this model renders it interesting to test p53-reactivating small molecules as a novel therapeutic approach [46]. Interestingly, we observed a strong expression of α-actin in non-treated tumor tissues, suggesting a dominant presence of cancer-activated fibroblasts in the cancer cell microenvironment. The tumor microenvironment comprises a variety of non-malignant stromal cells, providing support for tumor progression. If platinum-based compounds are known for their activity on gastric cancer cells, they may also impact the microenvironment [47]. For instance, Skolenova et al. described a relative resistance of human mesenchymal stromal cells to cisplatin treatment, together with changes in their secretory phenotype, compared to naïve ones in breast cancer [48]. In our study, we observed, by immunohistochemistry, changes in the tumor microenvironment (increased stroma/gland ratio) in the cisplatin treated group, which did not correlate with the presence of CAFs, with less dominant α-actin staining compared to the controls. These changes in the tumor microenvironment are confirmed by our RNA-Seq analysis, showing that cisplatin clearly induces the expression of genes associated with epithelial or mesenchymal origin. Published data showed that mesenchymal stromal cells are frequently found in the microenvironment of tumors [49]. In contrast, our data show that SAHA inhibits this mesenchymal signature, which is supported by findings of Xu and co-workers, showing that SAHA decreases the viability of mesenchymal stem cells, but stimulates osteogenesis [50]. Closely reproducing human tumors in terms of architecture and response to therapy, PDX models appeared to be the most accurate method to evaluate drug efficacy in preclinical studies.

Novel drug therapy development is crucial, especially in gastric cancers, where up to 75% resistance to platinum-derived chemotherapies have been described. For instance, one of the resistance factors against cisplatin in gastric cancer could be related to HDAC enzymes, especially HDAC4 [51]. HDAC enzymes are aberrantly expressed in various cancers including gastric cancer and HDAC expression is known to be linked to carcinogenesis. For this reason, HDAC inhibitors (HDACi) were developed to counteract their pro-oncogenic activity [13]. SAHA is one of the most promising HDACi, with an FDA approval for the treatment of cutaneous T-cell lymphoma. HDAC activity was clearly inhibited in our PDX model by the treatment with SAHA, as the analysis of the acetylation status of the lysine 9 of histone 3 (H3K9) showed an increased nuclear staining in the SAHA-treated tumors when compared to not treated ones. It is important to note that the increase in H3K9 acetylation is not specific to the cancer cells, but is also present in stromal cells. In addition, we observed some treatment-related morphological changes, such as cystic modifications of the tumors. Altogether, our results indicate that SAHA reduces tumor growth, as seen on the ultrasound measurement and Ki67 inhibition, confirming previous pre-clinical observation obtained on cell line-based models. Unfortunately, despite these promising preclinical data (i.e., inhibition of cellular growth and induction of apoptosis in cell-line-xenografts and confirmed by our data on PDX), SAHA failed phase 2 clinical trials in humans [16,52]. The exact cause of this failure remains unknown. Yet, our observations provide additional perspectives on the impact of HDAC inhibition on tumor structure, especially changes within the microenvironment, which will ultimately impact on the tumor response. Precise identification of the molecular relays involved in these structural changes will provide new insights for novel therapeutic combinations or for more accurate patients’ stratification, opening the way towards new imaging approaches on small animals in preclinical translational research.

In this regard, our transcriptional study of the cellular microenvironment of the tumors identified specific changes in gene expression corresponding to the activity of several transcription factors affected by cisplatin, SAHA, or both. For instance, HIF2alpha signature is induced by both treatments, although more importantly by SAHA. This is in accordance with the known regulation of HIF transcription factors by acetylation [15]. Interestingly, a signature for both MATH1 (also known as Atho1) and E47 is strongly induced by cisplatin in the tumor microenvironment. MATH1 has a complex role in the controlling cell proliferation and apoptosis in different organs (ex. intestine, cerebellum) [53]. Interestingly, these basic helix–loop–helix transcription factors, HIF2, MATH1 and E47, drive a growing interest for their role in the epithelial/mesenchymal transition. Hence, their activation by cisplatin and SAHA might account for some of the changes in the cellular identity signature observed in the microenvironment. It is also to be expected that each chemotherapy might also impact other cell types that may be present in the tumor microenvironment, such as neurons or tissue-specific cell types (e.g., endocrine cells). These cells may suffer from the chemotherapy and/or impact cancer cell survival via modulation of the activity of generic or specific transcription factors that control the expression of membrane or secreted proteins, defining a “secretome” able to transmit signal within the tumor ecosystem [54,55]. Additional studies will be required to fully apprehend how the activity of these transcription factors participate in the remodeling of the tumor microenvironment.

## 5. Conclusions

Altogether, this study shows how cisplatin and a pan-HDACi, SAHA, can both reduce tumor growth in a PDX model of gastric cancer with a similar amplitude. However, both drugs have a dramatically different impact on the microenvironment. In this regard, the use of an ultrasound is of great interest, as it allows following real-time evolution of the internal tumor structure in a cheaper and easier way, compared to alternative methods (i.e., MRI). The information gained does not impair the precision of the tumor measurement, and actually tends to improve it by allowing a 3D measurement and the conservation of imaging data, allowing the scientists to exchange data and redo measurements even after the image acquisition. Thus, this study might help to setup a novel standard for pre-clinical analysis of patient-derived xenografts when the antitumoral activity of metal-based compounds or other drugs has to be tested in vivo.

## Data Availability

All data can be directly obtained by contacting the authors.

## Supplementary Materials

The following are available online at www.mdpi.com/1999-4923/13/9/1485/s1. Figure S1. (a) Establishment of the PDX model (GXC004). (b) Detailed results of the NGS panel. (c) Example of clustering results obtained using AltAnalyze. Supplemental Methods: Genetic analysis, Sample preparation, method of mutation screening, gene list and data analyses of GCX004 tumor. Statistical analysis methods applied.
