# Supplementary material for: Ultrasound and Transcriptomics Identify a Differential Impact of Cisplatin and Histone Deacetylation on Tumor Structure and Microenvironment in a Patient-Derived In Vivo Model of Gastric Cancer
Source: Pharmaceutics. 2021 Sep 16;13(9):1485. doi: 10.3390/pharmaceutics13091485 (PMC8467189; doi:10.3390/pharmaceutics13091485)
Supplement: Supplementary file 1 [file pharmaceutics-13-01485-s001.zip › pharmaceutics-1346145-supplementary.pdf]

pharmaceutics MDPI

Supplementary Materials: Ultrasound and Transcriptomics Identify a Differential Impact of Cisplatin and Histone Deacetylation on Tumor Structure and Microenvironment in a Patient-Derived In Vivo Model of Gastric Cancer

Aina Venkatasamy, Eric Guerin, Anais Blanchet, Christophe Orvain, Véronique Devignot, Matthieu Jung, Alain C. Jung, Marie-Pierre Chenard, Benoit Romain, Christian Gaiddon and Georg Mellitzer

a

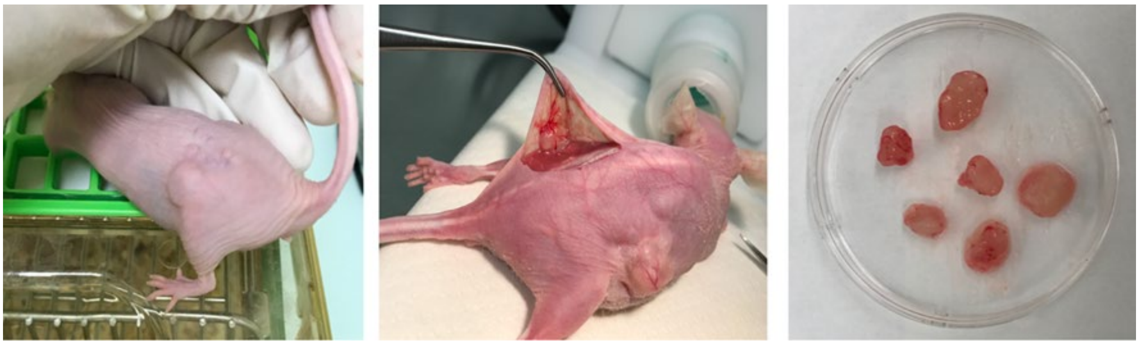

b

AKT1: NM_005163, exon 3
ALK: NM_004304, exons 20 à 29
BRAF: NM_004333, exons 11 et 15
CDKN2A: NM_000077 (p16/INK4a) et NM_058195 (p14/ARF), full coding sequence (exons 3)
CTNNB1 (bêta-caténine): NM_001904, exon 3
DDR2: NM_006182, full coding sequence (16 exons)
EGFR: NM_005228, exons 18, 19, 20 et 21
ERBB2/HER2: NM_004448, exons 19, 20 et 21
ERBB4/HER4: NM_005235, exons 10 et 12
FGFR2: NM_000141, exons 7, 12 et 14
FGFR3: NM_000142, exons 7, 9, 14 et 16
H3F3A: NM_002107, exon 2
HIST1H3B: NM_003537, exon 1
HRAS: NM_005343, exons 2, 3 et 4
IDH1: NM_005896, exon 4
IDH2: NM_002168, exon 4
KIT: NM_000222, exons 8, 9, 10, 11, 13, 14, 17 et 18
KRAS: NM_033360, exons 2, 3 et 4
MAP2K1/MEK1: NM_002755, exons 2 et 3
MET: NM_001127500, exons 2, 10, 14 (including splicing variants), 15, 16, 17, 18, 19 et 20
NRAS: NM_002524, exons 2, 3 et 4
PDGFRA: NM_006206.5, exons 12, 14 et 18
PIK3CA: NM_006218.3, exons 2, 3, 10, 11 et 21
PIK3R1: NM_181523.2, exons 11, 12 et 13
PTEN: NM_000314.6, full coding sequence (9 exons)
STK11/LKB1: NM_000455.4, full coding sequence (9 exons)

c

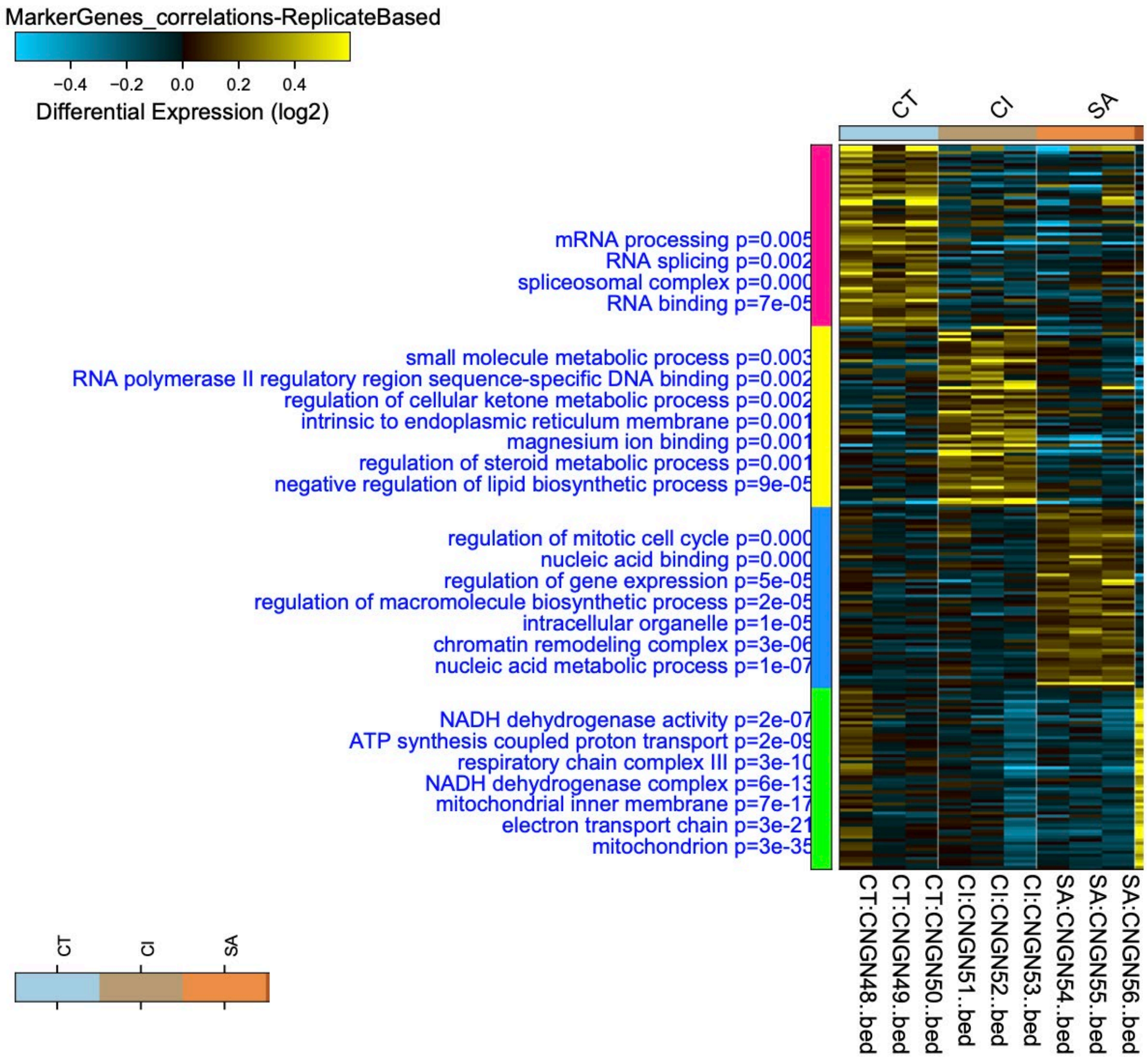

Figure S1. Supplementary data. (**a**) Establishment of the PDX model of gastric adenocarcinoma. The primary tumor sample was implanted subcutaneously on the flanks of the mouse (heterotopic implantation). When the tumor reaches the target volume ~1000mm3 (left image), the tumors were dissected (middle and right images) and 3 x 3mm fragments were prepared for serial implantation in the next generation of mouse. (**b**) Detailed results of the NGS panel of 26 clinically-targetable genes, showing no potentially pathogenic actionable mutation. (**c**) Results of genes' clustering using AltAnalyze software performed showing differentially regulated pathways between the experimental conditions. CT: control; CI: cisplatin; SA: SAHA.

Supplemental Methods:

Genetic analysis

DNA was extracted from the primary tumor from snap-frozen tissue and from PDX tumors using phenol/chloroform extraction protocol [18]. Mutation screening was performed on a MiSeq Illumina platform using Tumor Hotspot MASTR Plus assay (Multiplicom-Agilent). Sequencing data were aligned to human genome hg19 using BWA-MEM algorithm (Burrows-Wheeler Aligner-Maximal Exact Matches). Variants were called using three different variant callers: VarScan, GATK Haplotype Caller, and GATK Unified Genotyper. The minimum coverage per base and variant allelic frequency were fixed at 500-fold and 5% respectively. Data were visualized using the Integrative Genomics Viewer. A panel of twenty-six genes were analyzed (ATK1, ALK, BRAF, CDKN2A, CTNNB1, DDR2, EGFR, ERRBB2, ERBB4, FGFR2,

FGFR3, H3F3A, HIST1H3B, HRAS, IDH1, IDH2, KIT, KRAS, MAP2K1, MET, NRAS, PDGFRA, PIK3R1, PIK3CA, PTEN and STK11) together with the p53 mutational status.

Statistical analysis

Continuous variables were expressed as mean ± standard deviation (SD). Categorical variables were expressed in terms of numbers and percentages. Unpaired t-student test with equal SD was performed to compare the tumor volumes between the groups. The significance was set at $p = 0.05$. One-way ANOVA standard test was used for analysis of significance of the different measures (independent variables). Confidence level was chosen at 95% confidence-interval. Tukey's Multiple Comparison test was performed to compare caliper, ultrasound and gold standard. All statistical analyses were performed using GraphPad software 6.0.
